# Gross’ System of Surgery

**Published:** 1860-05

**Authors:** 


### BOOK AND PAMPHLET NOTICES.

Gross' System of Surgery. By Samuel D. Gross, M. D. Published by Blanchard & Lea, Philadelphia.

The high reputation of the author of this work on Surgery, together with its imposing and elaborate form, challenge for it a careful consideration. The work consists of two volumes of about twelve hundred pages each, and is divided into two parts. The first contains a discussion of the general topics of surgery, such as congestion, inflammation, tumors, wounds, etc. The second, and larger part, consists of special surgery arranged by organs, tissues, and regions. The whole is illustrated with about nine hundred wood cuts.

This work is intended to be the most complete system of modern surgery which has been published on this side of the Atlantic. For such a work Dr. Gross has some peculiar qualifications, as well as some minor defects. He has industry, a vast experience, and a power of grasping Titan-like at the summits of his subject without loosing himself in the discussion of unnecessary details. His style is sonorous and strong, but sombre and unmusical. It is disfigured also by a few unusual words, such as "timously" and "sakelessly," which are scarcely English, and not euphonic enough to deserve preservation in the language.

The great merit of the work may be stated as follows. It presents surgical science as it exists at the latest date, with all its improvements; and it discusses every topic in due proportion. Nothing is omitted, nothing is in excess.

Particular praise is due to the chapters on the surgery of the eye. In this department specialism has run mad. Authors have swelled their volumes with crude details, and distinguished themselves by the disgusting pedantry of a profession of useless technical terms, literally darkening counsel by words without knowledge. Excessive technicality indicates in a writer two grave defects—ignorance and a shallow intellect. It is the mark of a great mind to master a subject in such a way as to express it in simple language. This mark, Prof. Gross may justly claim. He has stripped away with a relentless hand the miserable collection of pet words which he found enveloping the subject, and by expressing himself in common surgical language, has restored this branch to its proper position. His bold simplification will greatly facilitate the study of the subject by under-graduates, and we hope lead practitioners to attend to their ophthalamic business as confidently and usefully as they do to fractures and dislocations, instead of turning it off to unprincipled quacks.

Whoever expects to find in this work brilliant displays of new discovery, or fine efforts at analysis and reasoning, will be disappointed. Dr. Gross' mind is remarkable rather for strength and cautious judgment than acuteness. The book before us is such as might be expected from such a pen. It omits nothing which is well established, and endorses nothing which is at all doubtful. It is therefore a safe and valuable guide, which will not lead the young surgeon into the pursuit of any wild theories, or doubtful practices.

